# Transitioning Beyond Mercury Sphygmomanometers: Diagnostic Accuracy and Agreement of Digital Blood Pressure Monitors in a South Indian Adult Population

**DOI:** 10.7759/cureus.114444

**Published:** 2026-08-12

**Authors:** Binu Areekal, Anju C Mathew, Manu Naish

**Affiliations:** 1 Department of Community Medicine, Government Medical College Ernakulam, Kochi, IND; 2 Department of Pulmonology, Government Medical College Ernakulam, Kochi, IND

**Keywords:** agreement, bland–altman analysis, blood pressure monitoring, diagnostic accuracy, digital bp monitor, hypertension, mercury sphygmomanometer, south india, validity

## Abstract

Introduction: Hypertension is a leading cause of morbidity and mortality, and its prevalence is increasing worldwide. Accurate blood pressure (BP) measurement is fundamental to clinical diagnosis and epidemiological surveillance, and any measurement errors can lead to substantial misclassification of hypertension, resulting in inappropriate clinical management and inaccurate estimates of disease burden. While automated digital monitors offer a practical alternative to manual devices, their real-world accuracy requires robust local validation during the phase-out of traditional instruments. The objective of the current study was to assess the validity and accuracy of a digital BP monitor against the gold-standard mercury device in an adult population in South India. Materials and methods: A cross-sectional diagnostic evaluation study was conducted among 262 adults attending the outpatient department of a teaching hospital in South India. Baseline BP was measured twice using the digital BP monitor (Omron HEM-7120, OMRON Healthcare Co., Ltd., Kyoto, Japan) with a one- to two-minute interval between readings. After a five-minute interval, BP was measured using a mercury sphygmomanometer (Novaphon, NOVAFON GmbH, Weinstadt, Germany). Validity, reliability, agreement, and bias were analyzed using paired t-tests, Pearson correlation, and Bland-Altman analysis. Results: Digital and mercury measurements demonstrated exceptionally strong linear correlations for systolic BP (r = 0.945) and diastolic BP (r = 0.845). The mean difference (digital minus mercury) was non-significant for both parameters: -0.167 ± 7.46 mmHg for systolic and 0.296 ± 6.63 mmHg for diastolic. When comparing the two devices, absolute differences within 5 mmHg were observed in 135 (51.5%) participants for systolic BP and 164 (62.6%) participants for diastolic BP. Categorical assessment showed substantial diagnostic agreement (Cohen’s kappa = 0.77), with a sensitivity of 84.0% and specificity of 92.9% for hypertension detection. Bland-Altman analysis revealed a negligible mean bias of −0.167 mmHg for systolic BP (95% limits of agreement (LoA): −14.79 to 14.46 mmHg) and 0.296 mmHg for diastolic BP (95% LoA: −12.70 to 13.29 mmHg). Conclusions: The automated digital monitor demonstrates good population-level agreement and diagnostic validity, making it a reliable tool for routine screening and epidemiological surveillance. However, given the wide limits of agreement, critical clinical decisions, especially in borderline cases, should be made with caution.

## Introduction

Hypertension or raised blood pressure (BP) is one of the leading risk factors for mortality, morbidity, and disability worldwide. An estimated 1.4 billion people, or 33.3% of adults aged 30-79 years, had hypertension globally in 2024 [1]. Elevated systolic BP contributed to an estimated 10.8 million deaths in 2019 [2].

In India, the prevalence of hypertension in adults has increased overall from 18.1% in the National Family Health Survey (NFHS)-4 to 22.8% in NFHS-5 (2021) [3]. Even in Kerala, a state with relatively better health indicators, studies report hypertension prevalence exceeding the national average. A recent cross-sectional study in the Ernakulam district, Kerala, found a high burden of hypertension, nearly 50%, indicating the urgent need for robust detection and management strategies [4].

Uncontrolled hypertension causes cumulative damage to target organs, leading to stroke, ischemic heart disease, heart failure, chronic kidney disease, retinopathy, and vascular dementia. Multiple studies from Kerala have reported a high prevalence of uncontrolled hypertension among those diagnosed, with proportions ranging from 54% to 68.3% [5,6]. The resulting health system burden, associated costs, mortality, and morbidity due to complications are substantial when hypertension is uncontrolled.

Accurate BP measurement is fundamental to the diagnosis, treatment initiation, and monitoring of hypertensive patients. Misclassification, either false positives or false negatives, can lead to inappropriate management. A false-positive classification can lead to unnecessary medication, psychological distress, and increased healthcare costs. On the other hand, a false-negative misclassification can lead to a missed opportunity to prevent complications and end-organ damage [7].

Precise BP measurement is critical, as minor errors can lead to dangerous over- or undertreatment. The clinical and public health impact of small variances is profound. A 5-mmHg reduction in SBP in a population is predicted to result in a 14% reduction in stroke death, a 9% reduction in coronary artery disease-related death, and a 7% reduction in total mortality [8]. A 5-mmHg difference in systolic BP corresponds to an estimated 6% absolute and 30% relative change in the prevalence of hypertension at the population level [9]. Thus, accurate measurement of BP is extremely important not only in clinical settings but also in epidemiological studies. Even a small error in measuring BP can have far-reaching clinical and epidemiological implications at both the individual and community levels.

The mercury sphygmomanometer has historically been the gold standard for indirect BP measurement, prized for its precision and stable calibration [10]. However, concerns over mercury’s environmental toxicity and disposal hazards led the United Nations Environment Program (UNEP) Minamata Convention to decide to phase out these devices by 2020 [11]. Nevertheless, India has sought an exemption until 2025 [12].

On the other hand, digital BP monitors offer many advantages: they are easy to use, operator-independent, portable, and suitable for widespread community, home, or hospital settings. A key drawback, however, is that their accuracy can vary significantly across devices, patient populations, and arm sizes.

There are limited data in the Indian context, particularly in Kerala, comparing specific digital devices with mercury sphygmomanometers in clinical and epidemiological settings. The objective of the current study was to assess the validity and accuracy of a digital BP monitor against the gold-standard mercury device in an adult population.

## Materials and methods

The current study, which assessed the validity and accuracy of a digital BP monitor, was conducted at Government Medical College, Ernakulam, a teaching hospital in Kerala, South India. This was a cross-sectional diagnostic test evaluation study. Data were collected from December 2024 to March 2025 from patients and bystanders who attended the outpatient departments of medicine and surgery of the hospital. The Institutional Ethics Committee of Government Medical College Ernakulam issued approval 29/24 dated January 6, 2025. As the study aimed to assess both the mean differences in systolic and diastolic BP and the diagnostic accuracy of the digital BP monitor compared to the mercury one, the sample size was calculated separately for both objectives, and the larger of the two calculated sample sizes was selected as the final sample size. The sample size needed to detect the diagnostic accuracy of a digital BP monitor was calculated using the formula

$$N = \frac{Z_{1-\alpha/2}^2 \cdot \mathrm{Sens} \cdot (1 - \mathrm{Sens})}{d^2 \cdot P}$$

where Sens is the sensitivity of the new test, Z1-α/2 is the desired confidence interval, and d is the precision. Here, sensitivity is 80% [11], d is 7%, and Z1-α/2 is 95%. Applying this in the formula and considering the prevalence of hypertension (P) as 48.2%, the required sample size of the study is n = 262. The sample size needed to detect the mean differences of systolic and diastolic BP between those measured using a mercury sphygmomanometer and a digital BP monitor was calculated using the formulas

$$N_{\mathrm{pairs}} = \frac{(Z_{1-\alpha/2} + Z_{1-\beta})^2 \cdot \sigma_d^2}{\Delta^2}$$

where µ1 is the pre-test mean, µ2 is the post-test mean, and Δ is µ2 - µ1, and

$$\sigma = \frac{\sigma_1 + \sigma_2}{2}$$

where σ1/ σ2/ is the standard deviation (SD) in pre-/post-test.

Here, the pre-/post-test means were taken as 77.9 and 79.9; the SD in the pre-/post-test was 9.4 and 10.7 [11]; the power was 80%, and the alpha error was 5%; the required sample size was found to be 200.

Since the sample size calculated for the first objective was larger, it was selected as the final sample size. All adult patients and bystanders over the age of 30 years in the medicine and surgery outpatient departments during the data collection period were included in the study. Those who were unwilling, pregnant women, and those whose BP could not be measured properly in the sitting position for any reason were excluded.

After obtaining informed consent from the participants, they were seated comfortably in a chair, and the basic sociodemographic information and clinical history were elicited using a structured interview schedule. BP measurements were taken after the participant had rested comfortably in a seated position for 15 minutes. For operational uniformity and to eliminate inter-arm physiological variance, all BP measurements, using both the digital monitor and the mercury sphygmomanometer, were consistently recorded from the right arm. A one- to two-minute interval was maintained between consecutive measurements obtained using an automated digital monitor (Omron HEM-7120, OMRON Healthcare Co., Ltd., Kyoto, Japan) to minimize the effects of repeated cuff inflation, including venous congestion, and to allow BP to stabilize before the subsequent measurement. Following a further five-minute rest period, BP was measured using a standard mercury sphygmomanometer (Novaphon, NOVAFON GmbH, Weinstadt, Germany).

During the procedure, the patient was instructed to sit with their feet flat on the floor, legs uncrossed, and their back supported. They were also advised not to talk or move to avoid interfering with the BP measurements. During the measurements, the arm was supported at the heart level, and appropriate cuff sizes were used over bare skin. When measuring BP using a digital BP monitor, all manufacturer instructions, including cuff positioning, were followed, and the average of the two readings was taken. Before each measurement using the mercury sphygmomanometer, “the mercury column was checked to ensure that it was at the zero level after full deflation. Korotkoff phase I and V sounds were taken as systolic BP and diastolic BP, respectively, for the mercury sphygmomanometer. Every day, the investigator checked the mercury column for bubbles. The cuff, bladder, inflation bulb, and valve were checked for smooth functioning and quality compliance.

The Omron HEM-7120 was selected because it is one of the most widely available and commonly used automated oscillometric upper-arm BP monitors in Indian clinical practice and home settings. Its widespread use supports evaluation of its diagnostic performance, which is particularly relevant to routine patient care and public health practice in India. Any participant who was newly diagnosed as hypertensive was taken to the medicine outpatient department for further management. Participants with previously diagnosed hypertension and normotensive participants were also provided with appropriate advice regarding hypertension control or prevention.

Data analysis

Data were entered in Excel (Microsoft Corp., Redmond, WA, USA) and analyzed using SPSS Statistics version 22.0 (IBM Corp. Released 2013. IBM SPSS Statistics for Windows, Version 22.0. Armonk, NY). Two consecutive measurements of systolic and diastolic BP obtained using the digital BP monitor were averaged for each participant and compared with a single reference measurement obtained using a standard mercury sphygmomanometer. Systematic bias between the two devices was assessed by calculating their mean difference (digital minus mercury) and testing it using a paired t-test. Agreement between the devices was evaluated using Bland-Altman analysis, with estimation of the mean bias and limits of agreement. To assess proportional and spectrum bias, measurement bias was compared across quartiles of mean BP using one-way ANOVA, and linear regression was performed to evaluate the association between measurement bias and BP level. The percentage distribution of absolute differences between systolic and diastolic BP readings obtained using the mercury and digital devices was also assessed. Sensitivity, specificity, positive predictive value, and negative predictive value were calculated to evaluate the diagnostic performance of the digital device. Cohen's kappa was computed to assess agreement in hypertension classification between the digital device and the reference standard. Pearson correlation analysis was performed to assess the linear association between BP measurements obtained using the two devices. A p-value of less than 0.05 was considered statistically significant.

## Results

Of the 262 participants in the study, ages ranged from 33 to 82 years, with a mean (±SD) of 49.3 ± 12.5 years. Over half of the participants, 133 (50.8%), belonged to the 40-60 years age group, while 71 (27.1%) were under 40 years and 56 (21.4%) were between 60 and 80 years. Regarding socioeconomic status, most participants belonged to the below poverty line category (152, 58.0%). Eighty-three participants (31.8%) had a prior diagnosis of hypertension, and 68 (25.9%) were currently receiving antihypertensive treatment. Basic demographic details of the study population are given in Table 1.

**Table 1:** Sociodemographic characteristics and hypertensive status of the study participants (N = 262)

Variable	Category	Frequency (n)	Percentage (%)
Age group	Less than 40 years	71	27.1
	40-60 years	133	50.8
	60-80 years	56	21.4
	>80 years	2	0.8
Gender	Male	112	42.7
	Female	150	57.3
Socioeconomic status	Below poverty line	152	58
	Above poverty line	110	42
Hypertension status	Previously diagnosed as hypertensive	83	31.8
	Currently on treatment	68	25.9

The mean systolic BP recorded using the mercury sphygmomanometer was 129.4 ± 21.8 mmHg, while the digital BP monitor recorded a mean systolic BP of 129.2 ± 21.5 mmHg. The mean difference between the two methods (digital minus mercury) was -0.167 ± 7.46 mmHg and was not statistically significant. A very strong positive correlation was observed between the two methods for systolic BP measurement (Pearson correlation coefficient r = 0.945).

Similarly, the mean diastolic BP measured using the mercury sphygmomanometer was 82.0 ± 12.0 mmHg, compared to 82.3 ± 11.7 mmHg using the digital device. The mean difference (digital minus mercury) was 0.296 ± 6.63 mmHg, and the difference was not statistically significant. A strong positive correlation was also observed for diastolic BP measurements (r = 0.845). The mean differences of systolic and diastolic BP and their statistical significance are given in Table 2.

**Table 2:** Mean differences in systolic and diastolic BP between mercury and digital BP monitors A p-value <0.05 is considered statistically significant. BP: blood pressure, SD: standard deviation, CI: confidence interval

Parameter	Mercury (mean ± SD)	Digital (mean ± SD)	Mean difference (digital minus mercury) mean ± SD	Paired t-test: t-value (p-value)	Pearson r (CI)
Systolic BP (mmHg)	129.4 ± 21.8	129.2 ± 21.5	-0.167 ± 7.46	-0.364 (0.716)	0.945 (0.925-0.953)
Diastolic BP (mmHg)	82.0 ± 12.0	82.3 ± 11.7	0.296 ± 6.63	0.722 (0.471)	0.845 (0.806-0.876)

Analysis of the absolute differences between the two devices showed that 135 (51.5%) of participants had systolic BP differences of less than 5 mmHg, while 229 (87.4%) had differences within 10 mmHg. Only five (1.9%) showed differences greater than 20 mmHg. For diastolic BP measurements, 164 (62.6%) had differences less than 5 mmHg, and 234 (89.3%) had differences within 10 mmHg. Differences greater than 20 mmHg were observed in only three (1.1%) of participants. The percentage distribution of absolute differences in systolic and diastolic BP readings between the mercury and digital devices is given in Table 3.

**Table 3:** Percentage distribution of absolute differences in systolic and diastolic BP readings between mercury and digital BP monitors BP: blood pressure

Difference between digital and mercury sphygmomanometers	Difference in systolic BP measurement			Difference in diastolic BP measurement		
	Frequency	Percent	Cumulative percent	Frequency	Percent	Cumulative percent
Less than 5 mmHg	135	51.5	51.5	164	62.6	62.6
5-10 mmHg	94	35.9	87.4	70	26.7	89.3
10-15 mmHg	18	6.9	94.3	18	6.9	96.2
15- 20 mmHg	10	3.8	98.1	7	2.7	98.9
>20 mmHg	5	1.9	100	3	1.1	100
Total	262	100		262	100	

Using the mercury sphygmomanometer as the reference standard, 94 (35.9%) of participants were classified as hypertensive (BP ≥140/90 mmHg), whereas the digital BP monitor identified 91 (34.7%) as hypertensive. The digital BP monitor correctly identified 79 hypertensive and 156 normotensive individuals.

The diagnostic agreement between the digital BP monitor and the mercury sphygmomanometer was substantial, with a Cohen’s kappa coefficient of 0.77 (CI: 0.694-0.854) and a p-value of 0.001. Concordant classification between the two methods was observed in 235 (89.7%) of participants, while the digital BP monitor misclassified 27 participants (10.3%) relative to the mercury sphygmomanometer. The digital BP monitor demonstrated a sensitivity of 84.0% and a specificity of 92.9% in detecting hypertension. The positive and negative predictive values were 86.8% and 91.2%, respectively. The positive likelihood ratio was 11.85, and the negative likelihood ratio was 0.17, indicating good diagnostic accuracy of the digital BP monitor in identifying hypertension. The validity and agreement of the digital BP monitor compared to the mercury one are shown in Table 4.

**Table 4:** Agreement and diagnostic accuracy of the digital BP monitor compared with the mercury sphygmomanometer BP: blood pressure

Digital monitor	Hypertension by mercury sphygmomanometer	Normotensives by mercury sphygmomanometer	Total
Diagnosed with hypertension by the digital BP monitor	79 (true positive)	12 (false positive)	91 (34.7%)
Classified as normotensive by the digital BP monitor	15 (false negative)	156 (true negative)	171 (65.3%)
Total	94 (35.9%)	168 (64.1%)	262 (100%)

**Figure 1:**
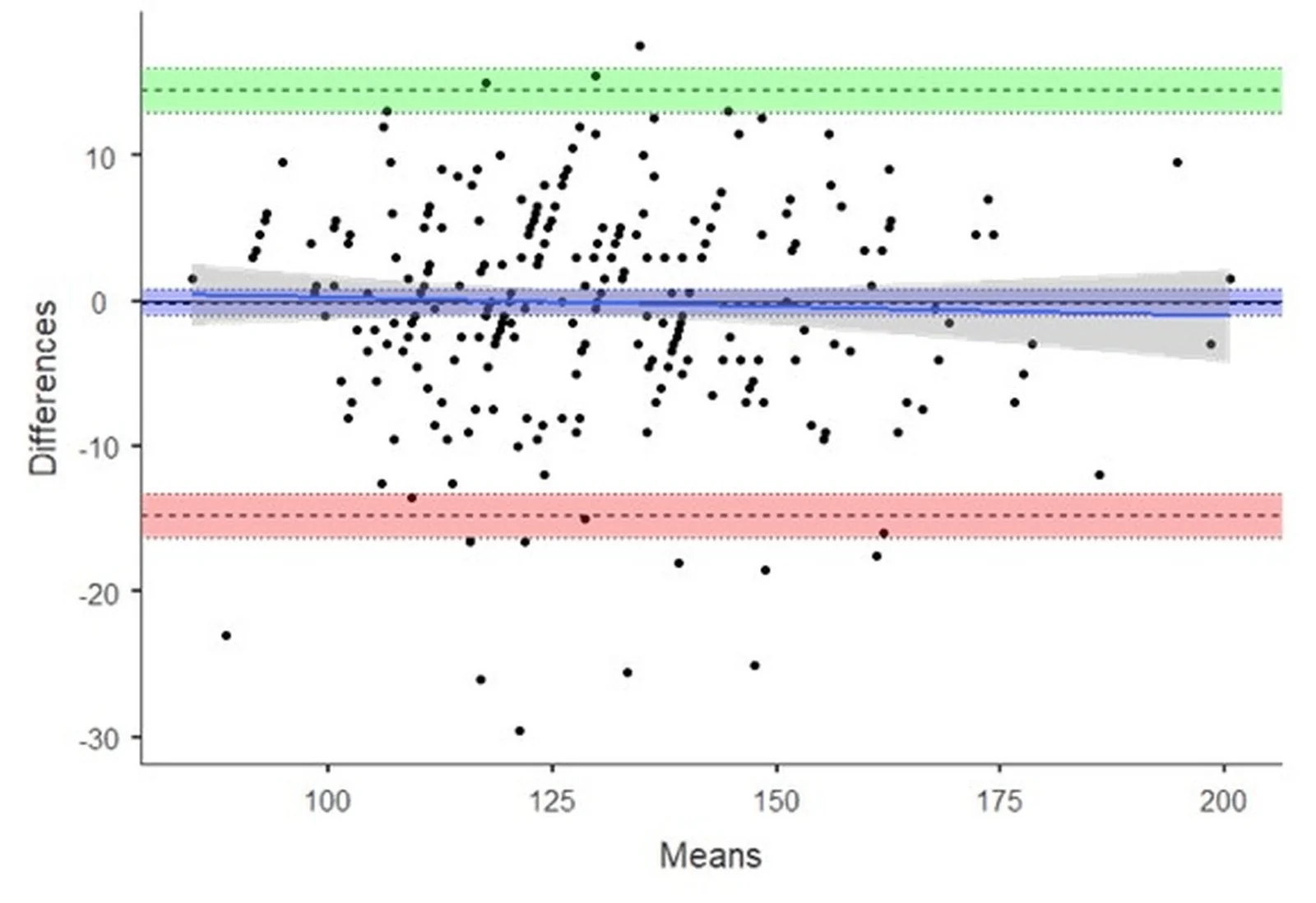
Bland-Altman plot of systolic BP measurements obtained using the digital BP monitor BP: blood pressure

**Figure 2:**
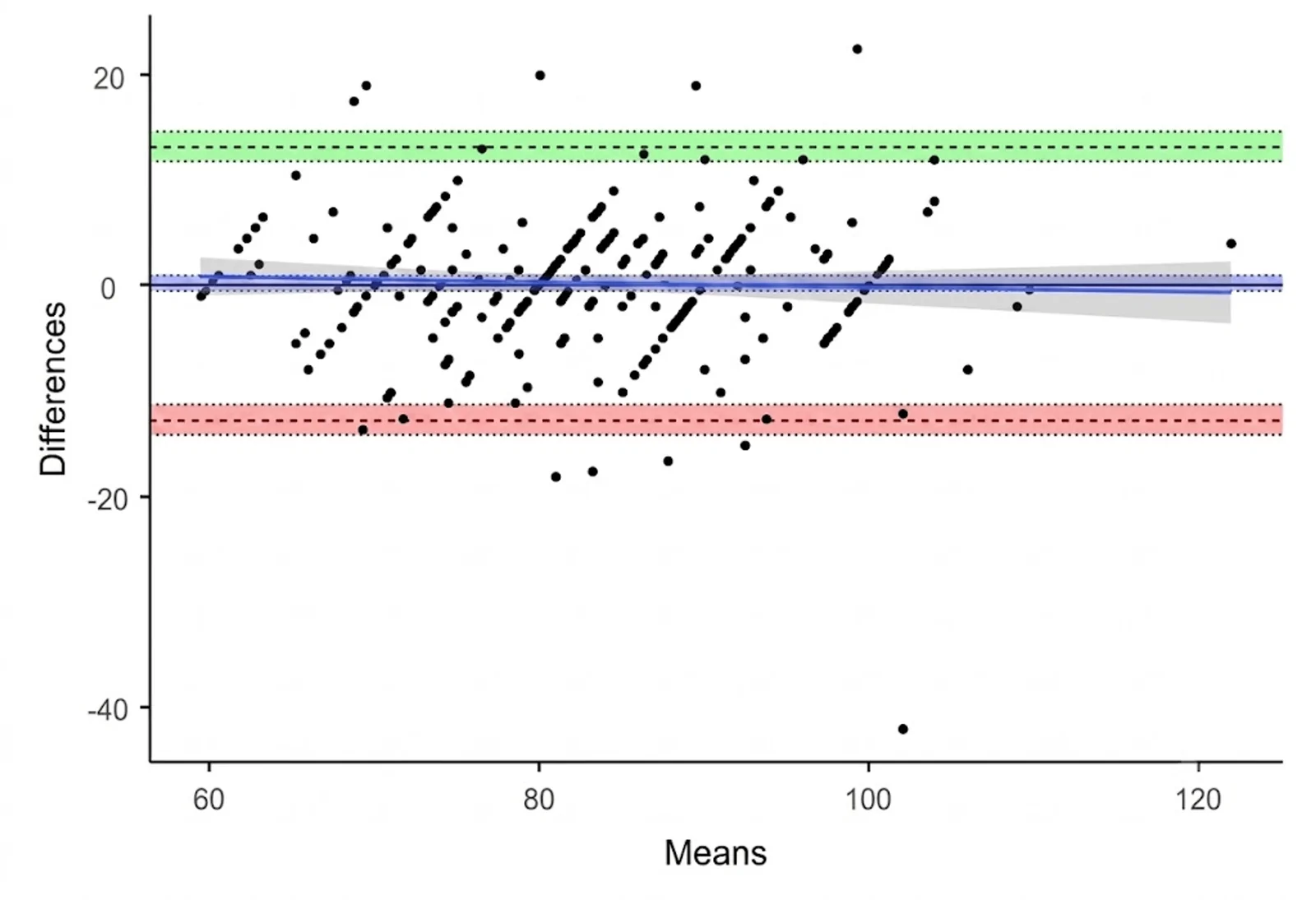
Bland-Altman plot of diastolic BP measurements obtained using the digital BP monitor BP: blood pressure

Bland-Altman analysis demonstrated good agreement between the digital BP monitor and mercury sphygmomanometer measurements. For systolic BP, the mean bias was −0.167 mmHg, with limits of agreement ranging from −14.79 mmHg to 14.46 mmHg (Figure 1). For diastolic BP, the mean bias was 0.296 mmHg, with limits of agreement ranging from −12.70 mmHg to 13.29 mmHg (Figure 2). Overall, Bland-Altman analysis demonstrated minimal systematic bias but relatively wide limits of agreement for individual measurements. To evaluate the possibility of spectrum and proportional bias across the BP range, measurement bias (digital minus mercury) was compared across quartiles of mean BP using one-way ANOVA, and linear regression was performed to assess the association between measurement bias and BP level. No significant differences in measurement bias were observed across systolic (ANOVA, p = 0.915) or diastolic (p = 0.697) BP quartiles. Similarly, linear regression showed no significant association between measurement bias and BP level for systolic (p = 0.560) or diastolic (p = 0.498) measurements, indicating no evidence of proportional or spectrum-related bias.

## Discussion

In the present study, the mean systolic and diastolic BP values recorded using the digital BP monitor were similar to those obtained with the mercury sphygmomanometer. The mean differences between the two methods (digital and mercury) were minimal and statistically non-significant for both systolic and diastolic BP measurements. Similar findings have been reported by Hamied et al. and Sreedharan et al., who observed no significant differences between BP readings obtained using digital and mercury devices [12,13]. The strong positive correlations observed for systolic BP (r = 0.945) and diastolic BP (r = 0.845) demonstrate a strong linear relationship between the values obtained from both instruments. A similar high correlation was reported by Ni et al. among 305 participants, with correlation coefficients of 0.89 for systolic BP and 0.81 for diastolic BP [14].

Furthermore, the observed agreement satisfies the World Health Organization technical specifications for automated non-invasive BP measuring devices with a cuff, which recommend a mean difference of less than 5 mmHg and an SD of less than 8 mmHg [15]. In our study, the mean difference was -0.167 mmHg with an SD of 7.46 mmHg for systolic BP and 0.296 mmHg with an SD of 6.63 mmHg for diastolic BP. These findings suggest that validated digital BP monitors can provide BP measurements comparable to those obtained using conventional mercury sphygmomanometers at the population level without systematic bias. However, the relatively high SD indicates variability in individual measurements.

Two hundred thirty-four (89.3%) diastolic BP measurements and 229 (87.4%) systolic BP measurements showed a difference of less than 10 mmHg, with only three (1.1%) demonstrating a difference greater than 20 mmHg for diastolic BP and five (1.9%) for systolic BP. These findings indicate a high level of categorical agreement between the two measurement methods. Varied observations have been reported in the literature evaluating the field performance of digital BP monitors. Choi et al. reported that 32.9% of systolic BP readings using digital BP monitors were within 5 mmHg and 61.1% were within 10 mmHg of mercury sphygmomanometer readings [16]. For diastolic BP, 22.2% were within 5 mmHg and 50.3% were within 10 mmHg. Similar conclusions have been reported by Hamied et al. and Sreedharan et al., who found that digital devices produced BP measurements within acceptable limits of agreement when compared with mercury sphygmomanometers [12,13]. Conversely, a study by Shahbabu et al., which evaluated the Omron HEM-7111 digital BP monitor, found a much lower level of conformity, with only 25.2% of systolic and 43.6% of diastolic BP readings differing by less than 5 mmHg from the mercury sphygmomanometer [11]. The superior rate of agreement observed in the present study may be attributed to our strict adherence to appropriate cuff size selection and the systematic minimization of observer bias, such as terminal digit preference, during manual mercury measurements.

Additionally, proprietary variations in oscillometric algorithms employed by different digital device manufacturers could also contribute to these differences. The strong correlations and minimal mean differences observed support the reliability and clinical utility of validated digital BP monitors for routine BP assessment in both hospital and community settings. Although most participants demonstrated acceptable agreement between the digital and mercury sphygmomanometers, measurement differences of 5-10 mmHg observed in a subset of participants may still be clinically relevant. While these differences had only a limited impact on the overall diagnostic agreement, they may lead to misclassification in individuals with BP values close to diagnostic or treatment thresholds.

The present study also demonstrated high diagnostic validity for the digital BP monitor in identifying hypertension, a critical parameter for both routine clinical practice and epidemiological screening. The device showed a sensitivity of 84.0%, specificity of 92.9%, positive predictive value of 86.8%, and negative predictive value of 91.2%. These findings are comparable to those reported by Ostchega et al. in the National Health and Nutrition Examination Survey (NHANES 2017-2018), which demonstrated a sensitivity of 86.3%, a specificity of 88.8%, and a Cohen’s kappa value of 0.75 for digital BP monitors [17]. Similarly, Singh et al. also reported high diagnostic performance of digital BP monitors in detecting hypertension [18]. In contrast, Shahbabu et al. reported a much lower sensitivity (80.0%) and specificity (67.0%) for digital devices when compared with a mercury sphygmomanometer [11]. The high diagnostic accuracy observed in the present study reinforces the suitability of validated digital BP monitors for hypertension screening and routine clinical use. Variations in diagnostic performance reported across studies may be attributable to differences in device calibration, measurement protocols, and observer expertise. Nevertheless, the overall evidence from the present study supports the use of validated digital BP monitors as reliable alternatives to mercury sphygmomanometers for both BP measurement and hypertension detection.

Unlike simple correlation, Bland-Altman analysis quantifies the measurement bias, revealing whether the digital monitor consistently over- or under-estimates BP across different measurement ranges, which is critical for clinical safety. In the current study, the mean measurement bias for digital BP monitors was negligible or close to zero, with limits of agreement (LoA) of −14.79 to 14.46 mmHg for systolic BP and −12.70 to 13.29 mmHg for diastolic BP. Similar results have been documented in the literature; for instance, Gogoi reported a minimal bias for systolic BP measurement of −1.6 mmHg with a LoA of −13.1 to 9.9 mmHg and a diastolic bias of −2.4 mmHg with a LoA of −20.4 to 15.5 mmHg for digital BP monitors [19]. Conversely, several other studies have reported more pronounced systematic errors. For example, Ahmed et al. reported larger mean biases ranging from −2.4 mmHg to +4.75 mmHg for both systolic and diastolic parameters, with limits of agreement extending from −15.0 to +24.7 mmHg for systolic BP and −14.0 to +21.0 mmHg for diastolic BP [20]. To further evaluate whether agreement varied across the BP spectrum, an additional analysis compared measurement bias across quartiles of mean BP and assessed the relationship between measurement bias and BP level using linear regression. Neither analysis demonstrated significant variation in measurement bias across the BP range, indicating no evidence of proportional or spectrum-related bias. Nevertheless, despite the absence of systematic or spectrum-related bias, the relatively wide limits of agreement indicate considerable variability in individual measurements. Therefore, although validated digital BP monitors provide comparable BP estimates at the population level, isolated digital readings may differ from mercury measurements by up to approximately 15 mmHg.

Ultimately, our study indicates that despite a near-zero mean measurement bias, the relatively wide limits of agreement suggest that individual digital BP readings may differ from mercury measurements by up to approximately 15 mmHg. Therefore, isolated digital measurements should be interpreted with caution, particularly when BP values are close to diagnostic or treatment thresholds and critical clinical management decisions are being made. Nevertheless, the negligible systematic bias, good diagnostic performance, and substantial agreement observed in this study support the use of validated digital BP monitors as practical alternatives to mercury sphygmomanometers for routine BP measurement and population-level hypertension screening.

Limitations

The study only evaluated one specific model of digital BP monitor (Omron 7120). Since different manufacturers use highly proprietary and varied oscillometric algorithms, the findings may not be generalizable to other digital BP monitor models. As a pragmatic protocol designed to minimize observer fatigue and prevent cumulative venous congestion from repeated cuff inflations on the same arm, a single mercury sphygmomanometer reading was used as the reference standard. In contrast, two consecutive digital readings were averaged, rather than the alternating measurement sequence recommended by international validation protocols. Although adequate rest intervals were maintained to minimize the effects of repeated cuff inflation and allow BP to stabilize, residual measurement-order bias cannot be completely excluded, even though our analysis showed a very low level of bias despite the fixed measurement order. Averaging two digital measurements while using a single mercury measurement as the reference may have reduced random variability in the digital measurements relative to the reference measurement, potentially influencing the agreement estimates. Although the study included participants with a broad range of BP values and detailed analyses using ANOVA and linear regression showed no significant spectrum bias, it cannot be completely ruled out, especially with very high BP values.

## Conclusions

 This study demonstrates that validated digital BP monitors offer a reliable and practical alternative to conventional mercury sphygmomanometers in clinical and epidemiological settings. However, the notable individual limits of agreement as revealed by the Bland-Altman analysis suggest that while these devices are useful for population-level surveillance and routine clinical practice, critical clinical decisions may benefit from manual verification to account for potential individual measurement differences.

Ultimately, given the ongoing global phase-out of toxic mercury-based instruments, these findings support implementing validated, well-calibrated automated monitors to preserve diagnostic accuracy while avoiding the environmental and health risks associated with mercury-based instruments.
